# Priorities in reducing child mortality: Azithromycin and other interventions

**DOI:** 10.1371/journal.pmed.1003364

**Published:** 2020-09-15

**Authors:** David Mabey, Uduak Okomo, Brian Greenwood

**Affiliations:** 1 Clinical Research Department, London School of Hygiene & Tropical Medicine, London, United Kingdom; 2 MRC Unit The Gambia at London School of Hygiene & Tropical Medicine, London, United Kingdom; 3 Disease Control Department, London School of Hygiene & Tropical Medicine, London, United Kingdom

## Abstract

In this Perspective, David Mabey and colleagues discuss a recent PLOS Medicine article on azithromycin as an intervention for reducing child mortality.

According to World Health Organization (WHO) estimates, the global under-5 mortality rate decreased by 59% between 1990 and 2018, from 93 to 39 deaths per 1,000 live births [1]. This remarkable reduction was achieved largely by increasing the coverage of vaccination against measles and other childhood infections, rolling out new vaccines against *Streptococcus pneumoniae* and *Haemophilus influenzae* type b, and introducing artemisinin combination treatment and long-lasting, insecticide-treated bed nets for the treatment and prevention of malaria.

In 2018, a randomised trial conducted in Niger, Tanzania, and Malawi—the Macrolides Oraux pour Réduire les Décès avec un Oeil sur la Résistance (MORDOR; “Oral macrolides to reduce death with an eye on resistance”) study—showed that twice yearly, community-based mass treatment with a single dose of azithromycin reduced all-cause under-5 mortality by 13.5% (95% CI 6.7–19.8) [2]. In children aged between 1 and 6 months, mortality was 24.9% lower in those who received azithromycin. Azithromycin is a long-acting, broad spectrum antibiotic that also has antimalarial, anti-inflammatory, and possibly antiviral properties [3,4]. At the study site in Niger, where the impact of azithromycin on mortality was greatest, verbal autopsy revealed fewer deaths from malaria, dysentery, meningitis, and pneumonia in communities treated with azithromycin, the prevalence of malaria parasitaemia was reduced, and the gut pathogen load was reduced after the fourth biannual azithromycin distribution [5–7]. Given that severe malnutrition increases the risk of life-threatening infectious diseases and is an important contributor to under-5 mortality, it seemed likely that the impact of azithromycin on mortality would be enhanced in malnourished children.

The paper by Kieran O’Brien and colleagues in this issue of *PLOS Medicine* investigates the question, comparing the impact of azithromycin mass treatment in malnourished (underweight) versus well-nourished children at the Niger study site of MORDOR [8]. The reduction in mortality in the azithromycin arm of the study (12.6 fewer deaths, 95% CI 6.9–18.5, per 1,000 person-years overall) was greater in malnourished than in well-nourished children, but not significantly so. The authors conclude that azithromycin mass treatment given to all children will have a greater impact on mortality than targeting treatment to malnourished children. While this is not unexpected, the potential impact of treating 10 times as many children on the antimicrobial susceptibility of important bacterial pathogens is an important consideration.

Bacterial infections remain a major cause of mortality among newborns [9] with *Staphylococcus aureus*, *S*. *pneumoniae*, and group B streptococcus (GBS) among the major pathogens [10]. Although the MORDOR trial excluded newborns, a recent study showed that administration of oral azithromycin during labour significantly reduced maternal and neonatal nasopharyngeal carriage of *S*. *pneumoniae*, *S*. *aureus*, and GBS as well as maternal and neonatal infections, with a short-term increase in the prevalence of azithromycin-resistant *S*. *aureus* among newborns of mothers who had received azithromycin [11,12].

A major target of the sustainable development goals is to end preventable deaths of newborns and children under 5 years of age by 2030, with all countries reducing under-5 mortality to less than 25 per 1,000 live births. While 120 WHO member states already meet this target, achieving it in all countries will be challenging. Whether and in what circumstances azithromycin mass treatment should be recommended remains debatable, and further research is needed to better understand the mechanisms by which the drug reduces mortality and the settings in which its impact is likely to be greatest while also evaluating the impact of this intervention on antimicrobial resistance.

What other interventions could help to achieve this target? Neonatal mortality has declined very little in the past 30 years, and almost 50% of childhood deaths are now in neonates, making interventions to reduce neonatal mortality and prevent stillbirths a high priority. More than 80% of all newborn deaths result from 3 preventable and treatable conditions—complications due to prematurity, intrapartum-related deaths (including birth asphyxia), and neonatal infections; a third of these deaths occur on the day of birth and nearly three-quarters in the first week of life. In 2014, WHO and UNICEF launched the Every Newborn Action Plan, which was endorsed by all 194 member states and which focuses on improving the quality of care around the time of birth [13]. It set out a clear vision of how to improve newborn health and prevent stillbirths. The target for 2035 is for all countries to reach the target of 10 or fewer newborn deaths and fewer than 10 stillbirths per 1,000 live births. How can this ambitious target be achieved?

Focusing on the critical periods before and immediately following birth is essential. The packages of care with greatest impact on neonatal deaths and stillbirths include care during labour, childbirth, and the first week of life and care for the small and sick newborn child. Recommended interventions include skilled care at birth, basic and comprehensive obstetric care (including parenteral administration of uterotonics, anticonvulsants, and antibiotics for preterm or prolonged rupture of membranes), management of preterm births (including the use of antenatal corticosteroids), a clean birth environment, and essential newborn care (i.e., hygienic care, thermal control, support for breastfeeding, and—if required—newborn resuscitation). It is important that the interventions for mother and newborn are seen as a functional unit delivered in a narrow time window by the same healthcare providers in the same place, with referral for management of complications including mother and baby together. These packages could, with universal access, prevent more than 1.9 million maternal and newborn deaths and stillbirths by 2025. The package of care for small and sick newborn babies includes interventions to deal with complications arising from preterm birth and/or small for gestational age and neonatal infections (sepsis, meningitis, pneumonia, and those causing diarrhoea), such as extra thermal care and support for feeding for small or preterm babies, including kangaroo mother care, antibiotic treatment for infections, and full supportive facility care. Focusing on small or sick newborns could prevent almost 600,000 newborn deaths by 2025 [14,15].

Antenatal care provides an opportunity for integrated service delivery for pregnant women, including obstetric services, and also provides the opportunity to treat and prevent malaria and syphilis in pregnancy, prevent mother-to-child transmission of HIV, and reduce harmful lifestyle practices such as smoking and alcohol use. Ensuring that all pregnant women are screened for syphilis using a point of care test, and that those who test positive are treated with a single dose of benzathine penicillin, would prevent an estimated 140,000 stillbirths and more than 60,000 neonatal deaths annually and is one of the most cost-effective health interventions [16,17]. Maternal immunisation also presents a specific opportunity to prevent neonatal infections, especially GBS. The use of antibiotics remains, therefore, an important intervention with a high impact on preventing stillbirths as well as reducing newborn and maternal deaths. However, increasing global antimicrobial resistance has the potential to erode the hard-won gains in maternal and newborn survival. Global commitment to antimicrobial stewardship is crucial to reverse this trend.

Mass treatment with azithromycin can reduce childhood mortality in some settings, but further research is needed to identify where it is likely to have an impact. Its blanket use to reduce childhood mortality remains fraught with unanswered questions [18]. Improvements in water, sanitation, and hygiene; provision of adequate health care; and improved coverage of immunisation and other effective interventions—as well as community participation in the planning, implementation, and monitoring of policies and programmes that affect them—remain the cornerstone of efforts to achieve health-related sustainable development goal targets.

## Abbreviations

GBS: group B streptococcus
MORDOR: Macrolides Oraux pour Réduire les Décès avec un Oeil sur la Résistance
WHO: World Health Organization

## References

[1] Under five mortality. Global Health Observatory (GHO) data. World Health Organization website. [cited 2020 Aug 21]. https://www.who.int/gho/child_health/mortality/mortality_under_five_text/en/.

[2] Keenan JD, Bailey RL, West SK, Arzika AM, Hart J, Weaver J et al Mass Azithromycin administration to Reduce Childhood Mortality in Sub-Saharan Africa.N Engl J Med. 2018; 378:1583–92. 10.1056/NEJMoa1715474 29694816 PMC5849140

[3] Sadiq ST, Glasgow KW, Drakeley CJ, Muller O, Greenwood BM, Mabey DCW et al Effects of azithromycin on malariometric indices in The Gambia. Lancet 1995; 346: 881–2. 10.1016/s0140-6736(95)92712-3 7564674

[4] Damle B, Vourvahis M, Wang E, Leaney J, Corrigan B. Clinical Pharmacology Perspectives on the Antiviral Activity of Azithromycin and Use in COVID-19. Clin Pharmacol Ther. 2020 4 17;10.1002/cpt.1857. 10.1002/cpt.1857 32302411 PMC7262099

[5] Keenan JD, Arzika AM, Maliki R, Adamou SE, Ibrahim F, Kiemago M et al Cause-specific mortality of children younger than 5 years in communities receiving biannual mass azithromycin treatment in Niger: verbal autopsy results from a cluster-randomised controlled trial. Lancet Glob Health 2020; 8: e288–e95. 10.1016/S2214-109X(19)30540-6 PMC7025321 31981558

[6] Arzika AM, Maliki R, Boubacar N, Kane S, Cotter SY, Lebas E et al Biannual Mass Azithromycin Distributions and Malaria Parasitemia in Pre-School Children in Niger: A Cluster-Randomized, Placebo-Controlled Trial. PLoS Med. 2019;16:e1002835 10.1371/journal.pmed.1002835 31237871 PMC6592520

[7] Doan T, Hinterwirth A, Worden L, Arzika AM, Maliki R, Abdou A et al Gut microbiome alteration in MORDOR I: a community-randomized trial of mass azithromycin distribution. Nature Medicine. 201925:1370–6. 10.1038/s41591-019-0533-0 31406349

[8] O’Brien KS, Arzika AM, Maliki R, Manzo F, Mamkara AK, Lebas E, et al Biannual azithromycin distribution and child mortality among malnourished children: A subgroup analysis of the MORDOR cluster-randomized trial in Niger. PLoS Med. 2020;17(9): e1003285 doi: 10.1371/journal.pmed.1003285 10.1371/journal.pmed.1003285 PMC7491708 32931496

[9] Levels & Trends in Child Mortality: Report 2019, Estimates Developed by the UN Inter-agency Group for Child Mortality Estimation. 2019. [cited 2020 Apr 5]. https://reliefweb.int/report/world/levels-and-trends-child-mortality-united-nations-inter-agency-group-child-mortality.

[10] Okomo U, Akpalu ENK, Le Doare K, Roca A, Cousens S, et al Aetiology of invasive bacterial infection and antimicrobial resistance in neonates in sub-Saharan Africa: a systematic review and meta-analysis in line with the STROBE-NI reporting guidelines. Lancet Infect Dis. 2019;19:1219–34. 10.1016/S1473-3099(19)30414-1 31522858

[11] Oluwalana C, Camara B, Bottomley C, Goodier S, Bojang A, et al Azithromycin in Labor Lowers Clinical Infections in Mothers and Newborns: A Double-Blind Trial. Pediatrics. 2017;139; e20162281 10.1542/peds.2016-2281 28130432

[12] Roca A, Oluwalana C, Bojang A, Camara B, Kampmann B, et al Oral azithromycin given during labour decreases bacterial carriage in the mothers and their offspring: a double-blind randomized trial. Clin Microbiol Infect. 2016;22:565 e1-9 10.1016/j.cmi.2016.03.005 27026482 PMC4936760

[13] Every newborn action plan. Maternal, newborn, child and adolescent health. ReachingWorld Health Organization website. [cited 2020 Aug 21]. https://www.who.int/maternal_child_adolescent/newborns/every-newborn/en/.

[14] Bhutta ZA, Das JK, Bahl R, Lawn JE, Salaman RA et al What will it take to avert preventable newborn deaths and stillbirths and at what cost? Lancet, 2014:384: 347–70. 10.1016/S0140-6736(14)60792-3 24853604

[15] World Health Organization recommendations on postnatal care of mother and newborn. Geneva: World Health Organization, 2013. 24624481

[16] Korenromp EL, Rowley J, Alonso M, Mello MB, Wijesooriya NS, Mahiané SG et al Global burden of maternal and congenital syphilis and associated adverse birth outcomes-Estimates for 2016 and progress since 2012. PLoS ONE. 2019;14:e0211720 10.1371/journal.pone.0211720 eCollection 2019. 30811406 PMC6392238

[17] Terris-Prestholt F, Watson-Jones D, Mugeye K, Kumaranayake L, Ndeki L, Weiss H et al Is antenatal syphilis screening still cost-effective in Sub-Saharan Africa? Sex. Transm. Infect. 2003;79:375–381 10.1136/sti.79.5.375 PMC1744759 14573832

[18] Tam CC, Offeddu V, Lim JM, Voo TC. One drug to treat them all: ethical implications of the MORDOR trial of mass antibiotic administration to reduce child mortality. J Glob Health. 2019;9:010305 10.7189/jogh.09.010305 30643634 PMC6318831

